# Pneumonic Plague Cluster, Uganda, 2004

**DOI:** 10.3201/eid1203.051051

**Published:** 2006-03

**Authors:** Elizabeth M. Begier, Gershim Asiki, Zaccheus Anywaine, Brook Yockey, Martin E. Schriefer, Philliam Aleti, Asaph Ogen-Odoi, J. Erin Staples, Christopher Sexton, Scott W. Bearden, Jacob L. Kool

**Affiliations:** * Centers for Disease Control and Prevention, Atlanta, Georgia, USA;; † Nyapea Hospital, Nebbi District, Uganda;; ‡ Centers for Disease Control and Prevention, Fort Collins, Colorado, USA;; § Uganda Virus Research Institute, Entebbe, Uganda

**Keywords:** Plague, disease outbreaks, pneumonia, plague transmission, research

## Abstract

The public and clinicians have long-held beliefs that pneumonic plague is highly contagious; inappropriate alarm and panic have occurred during outbreaks. We investigated communicability in a naturally occurring pneumonic plague cluster. We defined a probable pneumonic plague case as an acute-onset respiratory illness with bloody sputum during December 2004 in Kango Subcounty, Uganda. A definite case was a probable case with laboratory evidence of *Yersinia pestis* infection. The cluster (1 definite and 3 probable cases) consisted of 2 concurrent index patient–caregiver pairs. Direct fluorescent antibody microscopy and polymerase chain reaction testing on the only surviving patient's sputum verified plague infection. Both index patients transmitted pneumonic plague to only 1 caregiver each, despite 23 additional untreated close contacts (attack rate 8%). Person-to-person transmission was compatible with transmission by respiratory droplets, rather than aerosols, and only a few close contacts, all within droplet range, became ill.

## Abstract 2

In a case cluster, pneumonic plague transmission was compatible with respiratory droplet rather than aerosol transmission.

Naturally occurring plague occurs most frequently in bubonic or septicemic forms and is usually acquired through the bite of an infected rodent flea. Bubonic and septicemic plague are not transmissible from person to person, but if left untreated, plague bacteria can spread hematogenously to the lungs, resulting in secondary pneumonic plague. Pneumonic plague is contagious through infectious respiratory secretions, potentially resulting in direct airway infection (primary pneumonic plague) among close contacts (*1**,**2*).

Pneumonic plague epidemics in China early in the 20th century killed tens of thousands of persons (*3*). Plague is now rare in developed countries. However, the possibility of an intentional aerosol release of plague bacteria causing numerous contagious primary pneumonic plague cases has been a top concern of bioterrorism specialists (*1*). Consequently, *Yersinia pestis* release scenarios have been used in large-scale bioterrorism preparedness drills (*4**,**5*). The possibility of pneumonic plague importation's causing an outbreak in a nonendemic region is also a concern (*6*).

In-country panic and international alarm followed the 1994 report of pneumonic plague in India (*7*). Physicians reportedly fled Surat, the affected city, stating that there was "nothing to be done," and tetracycline was hoarded in areas distant from the reported outbreak (*7*). Some commercial airline flights (*8*) and exports (*7*) from India were cancelled. English physicians contested their public health officials' description of plague's low communicability based on their clinical training and infectious disease textbooks (*9*). Commercial repercussions for India have been estimated at US $3–$4 billion (*7*). Similarly and more recently, thousands fled a suspected pneumonic plague outbreak in the Congo during 2005 (*10*).

The public and clinicians have long-held beliefs that pneumonic plague is highly communicable (*9**,**11**–**13*). The current Infectious Diseases Society of America (IDSA) summary on *Y*. *pestis* as a bioterrorism agent notes secondary transmission risk is not well-quantified (*14*). Because of its rarity, recent published observations on its contagiousness are scarce, and few clinicians have first-hand knowledge of the disease.

We describe pneumonic plague's communicability and clinical course in a recently investigated cluster. On December 26, 2004, a Ugandan police officer telephoned a local physician (author G.A.) about a cluster of deaths in that country's West Nile region. The physician initiated an investigation that day and was joined the next day by US Centers for Disease Control and Prevention (CDC) staff who were in the area for a plague treatment trial.

## Methods

The surviving patient, caregivers and healthcare providers of the ill, and other close contacts of the deceased were interviewed to understand how the outbreak was propagated and to identify close contacts needing prophylaxis. The surviving patient's clinicians (G.A. and Z.A.) provided clinical information. Because CDC and the Ugandan Ministry of Health were conducting a plague treatment trial in the area, prospective enhanced surveillance for plague was already ongoing in the West Nile region, involving at least weekly local health center visits. For this enhanced surveillance, a probable bubonic plague case was defined as an illness with fever and tender lymphadenopathy without another cause for lymphadenopathy (e.g., cellulitis or streptococcal pharyngitis). For this cluster investigation, we conducted additional retrospective pneumonic plague surveillance by interviewing private drug shop owners, business owners, traditional healers, and other area residents. We defined a probable pneumonic plague case as respiratory illness of acute onset with cough producing grossly bloody sputum during December 2004 in Kango Subcounty, Nebbi District, Uganda. For this investigation, we defined a definite pneumonic plague case as a probable case with laboratory evidence of plague infection.

### Laboratory Methods

The surviving patient's sputum and serum samples were tested for direct and indirect evidence of plague infection. Sputum was placed on blood agar plates to recover live organisms, tested by polymerase chain reaction (PCR) for evidence of *Y*. *pestis* DNA, reacted with fluorescent-labeled antibody specific for *Y*. *pestis* and analyzed by fluorescent microscopy, and assessed for *Y*. *pestis* antigen by using 2 handheld immunochromatographic assays (i.e., dipsticks) (TetraCore, Inc., Gaithersburg, MD, USA, and New Horizons, Columbia, MD, USA). Serum samples collected during the acute phase of the disease were tested for antibody to F1 antigen of *Y*. *pestis* (*15*).

### Extraction of DNA and PCR

The genes *caf1*, *repA1*, and *pla* were analyzed by PCR. CDC has used these primers for many years for recognition of *Y*. *pestis* DNA. Primer sequences were *caf1*-f 5´-ATACTGCAGATGAAAAAAATCAGTTCC-3´, *caf1-r* 5´-ATAAAGCTTTTATTG GTTAGATACGGT-3´; *repA1-f* 5´-AGGCCCTGTTCACACATC-3´, *repA1-r* 5´-CCGGGTGTA GTTATTGTTCC-3´; and *pla-f* 5´-ATCTTACTTTCCGTGAGAA-3´, *pla-r* 5´-CTTGGATGTTGA GCTTCCTA-3´. Basic local alignment and sequencing tool analysis against all known sequences in GenBank demonstrated no significant homologies outside *Y*. *pestis* for *caf1* and *pla* primers. The *repA1* primer set also has 100% homology to *Y*. *pestis*, *Y*. *pseudotuberculosis*, and *Y*. *enterocolitica*. DNA was extracted from 200 μL of sputum by using the QIAamp DNA Blood Mini Kit (Qiagen, Valencia, CA, USA) and manufacturer's protocol. A total of 5 μL extracted DNA from the sputum or a positive control (*Y*. *pestis* strain CO 92) or negative control (water) was added to each reaction. PCR conditions were as previously described (*16*). Expected amplicon sizes were 531 bp (*caf1*), 833 bp (*repA1*), and 480 bp (*pla*). PCR was carried out by using puReTaq Ready-To-Go PCR Beads (Amersham Biosciences, Piscataway, NJ, USA).

### Direct Fluorescent-Antibody Test

Sputum was vigorously vortexed to disrupt the semisolid mass, then centrifuged at 8,000 × *g* for 5 min to pellet the solid material. The pellet was washed once in phosphate-buffered saline (PBS) and resuspended in 75 μL of PBS. Approximately 5 μL of concentrated sputum was used for direct fluorescent-antibody (DFA) microscopy as previously described (*17*) and visualized at 400× magnification.

## Results

### Cluster Description

We identified 1 definite and 3 probable pneumonic plague cases, comprising 2 concurrent index patient–caregiver pairs. We refer to the pairs as A and B, with numbers 1 and 2 designating index and caregiver cases within each pair, respectively. Index patient B1 became ill 1 day before cough onset in index patient A1. Despite extensive investigation, we identified no social links between these 2 index patients and no evidence of contact in the week before patient A1's illness onset. We identified no other illnesses clinically compatible with pneumonic plague occurring in December 2004 in Kango Subcounty by active surveillance. All case-patients' disease symptoms are shown by onset day in the Table. Overall, compared with index patients, caregivers' illnesses progressed more rapidly, including quicker bloody sputum onset (mean 1 vs. 6 days).

**Table:** National history and timing of symptom onset for index patients (secondary pneumonic plague) versus caregiver-patients (primary pneumonic plague), Uganda, December 2004

Symptoms†	Patient‡	Symptom onset by day of illness*																		
		1		2		3		4		5		6		7		8		9		
		AM	PM	AM	PM	AM	PM	AM	PM	AM	PM	AM	PM	AM	PM	AM	PM	AM		PM
Fever/chills	A1	X																		
	B1	X																		
	A2	O																		
	B2	O																		
Headache	A1	X																		
	B1	X																		
	A2	O																		
	B2	O																		
Lymphadenopathy	A1					X														
	B1	Unknown																		
	A2	None																		
	B2	None																		
Weakness	A1							X												
	B1				X															
	A2	O																		
	B2	O																		
Cough	A1									X										
	B1								X											
	A2	O																		
	B2		O																	
Chest pain	A1									X										
	B1												X§							
	A2	O																		
	B2	O																		
Productive cough	A1													X						
	B1									X										
	A2	O																		
	B2		O																	
Bloody sputum	A1															X				
	B1											X								
	A2				O															
	B2		O																	
Shortness of breath	A1																			
	B1											X								
	A2			O																
	B2			O																
Nonambulatory	A1																			
	B1													X						
	A2					O														
	B2	Never																		
Death/effective																		X¶		
treatment	B1														X¶					
	A2						O¶													
	B2				O#															

*X, secondary pneumonic plague index patient; O, primary pneumonic plague caregiver-patient. AM, symptom first appeared between midnight and noon; PM, symptom first appeared between noon and midnight. †Additional symptoms: caregiver A2 had a sore throat and ulcerative pharyngitis. Caregiver B2 had a burning back pain that started at the time of illness onset. Index patients A1 and B1 both had vomiting, and B1 also had diarrhea. ‡Cases are listed by time of onset: 12/9 (A1), 12/13 (B1), 12/23 (A2), 12/25 (B2). Caregiver A2 reported her husband was ill about midday on 12/23, but symptoms might have started earlier. Caregiver A2 was index patient A1's mother and her primary caregiver during her illness. Caregiver B2 was index patient B1's sister and his primary caregiver during his illness. §Patient B1 first reported to family members severe chest pain on day 6 of illness but might have had mild chest pain earlier. ¶Death. #Treatment and survival.

Index patient A1 was a 22-year-old woman, and her primary caregiver, patient A2, was her 40-year-old mother. According to family members, patient A1's illness began with several days of headache, fever, and chills. Lymphadenopathy was first observed on day 3. Coughing, first noted on day 5, became productive a day later and bloody sputum was noted on day 7. On day 6, she was seen by a drug shop owner (a government-trained nursing assistant) and treated for malaria with 3 days of chloroquine. On day 9, she was coughing frank blood and died later that night. Patient A1's primary caregiver, patient A2, became ill 5 days after her daughter's death. On the first day of patient A2's illness, she reported headache, fever, chills, weakness, chest pain, and a productive cough. The same private drug shop owner examined her and reported ulcerative pharyngitis, a sign associated with inhalational exposure to *Y*. *pestis* (*12*), but not lymphadenopathy. The patient was treated with intramuscular penicillin (6 treatments over 36 h) for presumptive severe pneumonia. The next day grossly bloody sputum developed, and she died on illness day 3.

Index patient B1 was a 25-year-old man, and his primary caregiver, patient B2, was his 30-year-old sister. Index patient B1's illness began with headache, fever, and chills. His family sought care for him at a private drug shop and transported him to 3 government health centers. Lymphadenopathy was not reported, although his clinicians did not specifically examine him for it. He received antimalaria treatment and intramuscular penicillin for presumptive severe pneumonia. His cough became productive with bloody sputum on day 5 of illness, and he died on day 6. Patient B1's primary caregiver, B2, became ill 5 days after patient B1's death.

Surviving caregiver B2 was identified the day the outbreak was reported, a day after her illness onset. She was markedly dyspneic, ill-appearing, with an elevated oral temperature and respiratory rate (39.3°C and 56 breaths/min, respectively). She required assistance to walk. She had no palpable lymph nodes. A pulmonary examination showed marked chest indrawing and bilateral coarse crepitations. She was first treated 29 h after illness onset at the local health center, where she received chloramphenicol, 2 g intravenously as a single bolus, and doxycycline, 100 mg orally.

**Figure 1:**
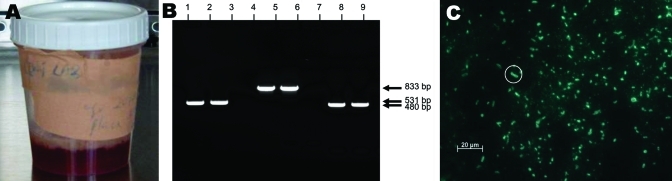
A) Grossly bloody sputum sample obtained from the surviving patient (caregiver B2) 30 h after onset of primary pneumonic plague. B) Polymerase chain reaction results of sputum sample from caregiver B2. Lanes 1–3, *caf1*; lanes 4–6, *repA1*; lanes 7–9, *pla*. Lanes 1, 4, and 7 are positive controls; lanes 2, 5, and 8 are patient samples; lanes 3, 6, and 9 are negative controls. C) Anti-F1 direct fluorescent antibody staining of sputum sample from caregiver B2. Numerous bacteria with classic halo structures are characteristic of *Yersinia pestis*. The circled bacterium classically depicts this halo.

On her arrival at Nyapea Hospital, a grossly bloody sputum sample was obtained (Figure 1A). Because hospital staff were unaware of her previous treatment, she was retreated with chloramphenicol, 1 g intravenously, given 1 h and 45 min after her initial dose. She continued treatment with chloramphenicol, 1 g intravenously every 6 h for 48 h, then 750 mg every 6 h (10 days of intravenous treatment). She was discharged at day 10 and received oral chloramphenicol, 750 mg every 6 h for 8 additional days. On discharge, she was able to walk 1 mile to her home from the nearest road but with difficulty and shortness of breath. Three weeks after being discharged, she reported having returned to all usual activities including subsistence farming.

**Figure 2:**
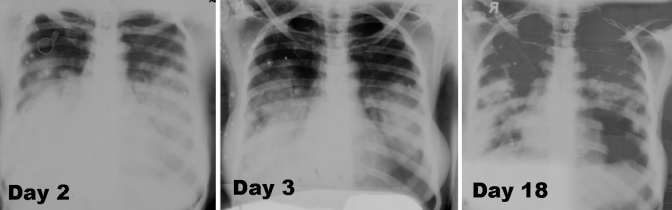
Three serial frontal chest radiographs from surviving caregiver B2 with primary pneumonic plague obtained on illness days 2, 3, and 18 showing bilateral lower lung zone predominant airspace disease associated with bilateral (right > left) pleural effusions. The radiographs have artifacts related to hand-dipping of the films, which account for multiple densities that move between images and the areas of apparent lucency.

A series of 3 frontal chest radiographs taken on days 2, 3, and 18 of illness demonstrated bilateral airspace disease, predominantly in lower lung zones, with bilateral (right > left) pleural effusions without evidence of hilar or mediastinal lymphadenopathy (Figure 2). Findings were consistent with multilobar pneumonia with progressive diminution in airspace disease and pleural effusions over time.

Presence of *Y*. *pestis* in the surviving patient's sputum was verified by positive PCR results for genes on all 3 of the *Y*. *pestis* plasmids (Figure 1B), and DFA showing classic fluorescent staining halos of bacteria with *Y*. *pestis*-specific antibody (Figure 1C). Two handheld assays also detected *Y*. *pestis* antigen in the sputum. The sputum, which was obtained 1.5 h after her first antimicrobial drug dose, stored overnight without refrigeration, and transported the next day to the central laboratory (6 h in transport), did not yield *Y*. *pestis* on bacterial culture. A complete blood count at illness day 20 was within normal limits. Antibody to *Y*. *pestis* was not detected in serum from acute-phase blood samples. The patient declined to provide a sample for convalescent-phase serologic testing at a follow-up visit 3 weeks after discharge. The other 3 case-patients were already buried when the outbreak was reported; therefore, autopsies and laboratory verification of their plague diagnoses were not attempted.

### Contact Tracing and Prophylaxis

Close contacts of index patients A1 and B1 are described in the Table A1. These contacts were not given antimicrobial drug prophylaxis because >1 week had passed since the index patients' deaths when the outbreak was reported. Twenty-five persons had direct contact with either patient (i.e., touched) after onset of cough productive of bloody sputum and before death, but only the 2 primary caregivers became ill (attack rate 2/25, 8%).

Examples of these index patients' close contacts include the following. Patient A1 slept in the same bed as her husband and 1.5-year-old daughter in a 1.8 × 3.1 × 2.0 m bedroom the night before her death. The night before index patient B1's death, he slept in the same bed as his 6-year-old daughter until the early morning, when his wife noted he was very ill and coughing bloody sputum. His daughter then moved to a straw mat on the floor of the 4 × 4 × 1.6 m windowless 1-room house with her mother and 3 siblings, who had been sleeping there. Their heads were ≈1.8 m from their father's.

On index patient B1's last day, he was placed in a chair strapped on the back of a bicycle and transported 18 km to obtain medical care at several clinics. His 3 brothers who held him upright during this trip remained well without prophylaxis. In addition, ≈200 persons attended the 2 index patients' funerals; ≈75 persons touched the blanket that wrapped index patient B1's body, the same blanket that was used during the patient's final days of illness. No contacts used masks, gloves, or any other form of respiratory protection.

All identified close contacts of caregivers A2 and B2 received chemoprophylaxis (3 days of cotrimoxazole, 960 mg orally, twice a day), including 14 members of caregiver A2's family compound, 8 members of caregiver B2's family compound, and 4 healthcare providers who rode without masks in the ambulance with caregiver B2. Prophylaxis was initiated 4 days after caregiver A2's death, 2 days after caregiver B2's treatment initiation, and on the day of the ambulance ride, respectively. Additionally, local health authorities gave prophylaxis to 200 attendees of caregiver A2's funeral on the day of the funeral, which took place the morning after A2's death, the same day the outbreak was reported.

### Community Surveillance

No additional pneumonic plague cases were identified during December and in the weeks after the outbreak report. However, through active surveillance we identified 3 probable bubonic plague patients who came to the subcounty's local health center in the first half of January, an increase from a baseline of 0 cases per month in the preceding 3 months. In addition, during the investigation in late December and early January, several villages in the subcounty reported rat deaths, and both index patients' families reported finding dead rats in their family compounds before the index patients' illness onset.

## Discussion

We report 4 pneumonic plague cases involving 2 instances of person-to-person transmission. Even without appropriate treatment, the 2 index patients survived >1 week. The index patients transmitted pneumonic plague, likely in their final hours of life, to only their primary caregivers, despite numerous other close contacts. This transmission pattern is compatible with respiratory droplet transmission rather than transmission by aerosols (droplet nuclei). Furthermore, only a few close contacts, who were all within droplet range, became ill. Primary pneumonic plague developed in the primary caregivers, who displayed a more fulminant clinical course. However, 1 survived without residual functional limitation after chloramphenicol treatment initiated 29 h after illness onset, which is later than commonly thought useful (*11**,**12**,**14*).

We identified 23 close contacts of the 2 index patients who remained well without antimicrobial drug prophylaxis or other form of protection, including 3 family members who slept in the same bed and many persons who slept with their heads at a distance <2 m from the coughing plague patient. Some published literature describes pneumonic plague as highly contagious (*9**,**12*) through aerosols (droplet nuclei) (*13*). However, other researchers have reported that transmission requires prolonged close contact at the end stage of illness (*2**,**18**,**19*), which is consistent with respiratory droplet transmission (*1**,**20*). This investigation supports the latter view. Furthermore, droplet range is usually <3 feet (*21*), and all identified close contacts were well within that proximity to an index patient, but few (8%) became ill. Transmission likely occurred on the index patients' final day of life, given the 5-day interval until caregiver symptom onset after the index patients' death (incubation period usually 2–4 days, range 1–6) (*2**,**19*). Consistent with our findings, Gani and Leach's review of pneumonic plague outbreaks reported an average 1.3 pneumonic plague transmissions to other persons per pneumonic plague case (*22*). An investigation of a larger outbreak in Madagascar that used serologic testing to confirm plague infections also reported an attack rate among close contacts similar to ours (8.4%), although a definition of close contacts was not reported (*23*).

Our patients' clinical course provides clues to why pneumonic plague patients usually infect few persons and why, for example, an air travel–associated outbreak would be unlikely. Our case-patients were visibly short of breath, coughing grossly bloody sputum, and barely ambulatory before transmitting the disease. Thus, when patients are substantially contagious, they are unlikely to be traveling by air and, if so, would appear ill enough to alarm nearby passengers. In most settings, persons this ill are at home or in the hospital. Recent reviews support this observation because most reported pneumonic plague transmissions involve family, friends, or medical professionals caring for ill persons at home or in the hospital (*2**,**22*).

A current IDSA summary on *Y*. *pestis* as a bioterrorism agent notes, "in the absence of early therapy (i.e., within the first 24 h), death occurs from overwhelming sepsis" (*14*). This follows Butler's widely cited reviews, which state that pneumonic plague is "invariably fatal" if treated >20–24 h after illness onset (*11**,**12*) and cite the 1956 report of McCrumb et al. (*24*). More recent reviews (*25*) and other literature (*26**,**27*) indicate that survival is possible even when treatment is initiated after 24 h, consistent with caregiver B2's survival. This caregiver received chloramphenicol, the only parenteral drug designated as a national standard plague treatment in Uganda (*28*), 29 h after illness onset, and survived without supportive care (i.e., mechanical ventilation or oxygen therapy). In the United States, chloramphenicol is a second- or third-line plague treatment (*1**,**29*) because no randomized clinical trials have been conducted to document its comparability with accepted treatments and because it has potential hematologic side effects (*1*). Although caregiver B2 received supratherapeutic doses because of a communication error, experienced Ugandan clinicians report success treating plague, including pneumonic plague, with chloramphenicol, 500 mg intravenously every 6 h (*30*).

Clinical diagnosis of pneumonic plague is challenging, particularly without lymphadenopathy. Even in this plague-endemic area these cases were not suspected to be plague until an investigation was initiated after the third death. All 4 cases had 1 classic pneumonic plague feature: productive cough progressing to grossly bloody sputum (*2**,**18**,**19*). In plague-endemic regions, respiratory illnesses <1 week in duration with bloody or blood-tinged sputum should prompt consideration of a pneumonic plague diagnosis and empiric antimicrobial drug treatment for plague. Routine chest radiographs cannot be expected to establish a pneumonic plague diagnosis. The chest radiographs of surviving caregiver B2 were consistent with but not uniquely diagnostic for primary pneumonic plague. As with the few other published radiographs of primary pneumonic plague patients (*1**,**24**,**27**,**31**–**33*), these radiographic findings alone would not prompt clinicians to consider pneumonic plague without a preexisting clinical suspicion.

In our cluster, primary pneumonic plague (direct airway infection) progressed rapidly to life-threatening illness. In contrast, respiratory symptoms developed later in illness for the apparent secondary pneumonic plague patients, consistent with hematogenous spread from an alternate site of infection initiation, and their symptoms progressed more slowly. The more fulminant clinical course of primary pneumonic plague could help differentiate primary versus secondary pneumonic plague in naturally occurring outbreaks and pneumonic plague suspected of being caused by an intentional bacterial release because aerosol exposure would result in primary pneumonic plague. Time course of clinical progression can be established retrospectively from history alone, in contrast to lymphadenopathy (a bubo), which can also help differentiate primary and secondary pneumonic plague but requires a thorough physical examination. A bubo indicates that pneumonic plague is most likely secondary to a primary bubonic plague infection (*14*).

Upon hearing of 4 cases in close proximity, our initial assumption was these cases were linked. However, closer investigation demonstrated that the second patient became ill before the first patient developed cough, and these 2 patients had no apparent contact during the week before the second patient's illness onset. They lived within 2 km of each other but were from different villages and tribal backgrounds. This coincidence indicates the importance of detailed contact investigations before pneumonic plague cases are declared linked in areas with ongoing epizootics. The cluster is likely explained by the annual epizootic reaching the area and difficulty diagnosing pneumonic plague. Because the 2 index patients lived near each other, they were likely both exposed to the same epizootic (i.e., plague-infected rats and fleas). Since their illnesses went unrecognized and were not appropriately treated, the patients each transmitted their infection to their caregivers, creating this 4-case cluster.

Among the investigation's limitations, we depended on family members' recall for information on deceased patients' symptoms and activities. However, we believe multiple family member interviews and rapid investigation initiation minimized information loss. Another limitation was our inability to culture *Y*. *pestis* from the surviving patient's sputum, which likely resulted from administration of an antimicrobial drug before sputum collection and suboptimal specimen storage and transport. However, we verified plague infection by laboratory analysis of this sputum sample, including amplification of all 3 targeted plague plasmids by PCR and visualization of numerous bacteria with classic halos of *Y*. *pestis* by DFA staining. Additionally, the sputum tested strongly positive with both *Y*. *pestis* antigen dipsticks. Finally, we could not verify all cases through laboratory analysis because 3 case-patients had been buried by the time the outbreak was reported. However, high death rate, fulminant clinical course, laboratory verification for the surviving case, and clinical symptoms were consistent with plague. The concomitant bubonic plague increase and reports of rat deaths provide additional support that plague was endemic during this outbreak.

In conclusion, this investigation illustrates the clinical course of pneumonic plague, contrasts secondary and primary disease, and shows the relatively low communicability of pneumonic plague even with numerous close contacts. This information should guide bioterrorism response planning and the public health response to naturally occurring pneumonic plague outbreaks.

## Appendix

**Table A1:** Summary of contacts of index patients A1 and B1 after onset of bloody sputum

Relationship to patient (person count)	Exposures					
	Touched patient	Handled excreta or sputum	Patient coughed near face	Listened to patient whispering	Exposure description	Outcome plague illness
Index patient A1*						
	Mother (1)	Yes	Yes	Yes	Yes	Primary caregiver during illness; washed body after death.	Case A2
	1.5-y-old daughter (1)	Yes	No	No	No	Slept facing A1 (chest level) for 1 night in same bed, the night before A1's death.	No
	Husband (1)	Yes	No	No	No	Slept in same bed facing away from A1 the night before her death; bicycled her to traditional healers; checked on her at healer's home in afternoon for ≈1 h.	No
	Traditional healer (1)	Yes	Yes	No	No	A1 spent her last day at healer's home, and healer buried bag of her bloody sputum. Provided A1 with tea and porridge. Spoke with her at a distance <2 m.	No
	Father (1)	Yes	No	No	No	Spent several hours with A1 during last day; spoke with her at a distance <2 m.	No
	Other male relatives (4)	Yes†	No	No	No	Put A1's body in casket; washed hands afterwards.	No
	Funeral attendees (50)	No	No	No	No	Viewed A1's body in casket; no one touched body.	No
Index patient B1‡						
	Sister (1)	Yes	Yes	Yes	Yes	B1's primary caregiver during illness after bloody sputum noted, including during transport.	Case B2
	Brother (1)	Yes	Yes	No	Yes	Oversaw B1's transport on bicycle for care; assisted with care; washed B1's body after death	No
	Wife (1)	Yes	No	Yes	Yes	B1's primary caregiver before bloody sputum and afterwards assisted primary caregiver before B1 left family compound; slept in same hut as B1 the night before death with her head 1.8 m from that of B1.	No
	6-y-old daughter (1)	Yes	No	No	No	Slept in bed with B1 until too ill night before death, then slept with siblings as follows.	No
	Other children (3)	Yes	No	No	No	Slept in same hut as B1 night before death with their heads 1.8 m from that of B1.	No
	Other female attendants (8)	Yes	No	No	No	Assisted in caring for B1 before his departure for clinics	No
	Brothers 2 and 3 (2)	Yes	Yes	No	No	Assisted in keeping B1 upright while he was being transported on a bicycle to clinics; 1 helped wash body after death.	No
	Neighbor (1)	Yes	No	No	Yes	Friend of patient; listened to B1 speak very close to his face before departure to clinics.	No
	Nursing assistants at 2 clinics (2)	Yes	No	No	No	Assessed and treated patient, including multiple intramuscular injections.	No
	Funeral attendees (150)	No	No	No	No	75 touched blanket that wrapped B1's body to view face; same blanket was used during final days of illness.	No

*Bloody sputum developed in patient A1 on December 16, 2004, and she spent that night at her home in the same bed as her husband and child. She rode seated on the back of bike to the traditional healer's home in the morning. She worsened over the course of the day and was coughing frank blood by the afternoon. She died that night at the healer's home while being cared for by her mother (A2). †Only touched patient after death, not included in close contacts. ‡Bloody sputum developed in patient B1 in the early morning of December 18, 2004. B1's daughter was moved out of his bed to sleep with his wife and their other children in the same hut with their heads ≈1.2 m from his. His sister (B2) came in and began caring for him. At dawn, B1 was placed on a chair on the back of a bicycle and transported to 3 clinics in succession (1 was closed). B1 died that night at a relative's home shortly after he arrived there.
